# Loss of Expression of Reprimo, a p53-induced Cell Cycle Arrest Gene, Correlates with Invasive Stage of Tumor Progression and p73 Expression in Gastric Cancer

**DOI:** 10.1371/journal.pone.0125834

**Published:** 2015-05-08

**Authors:** Kathleen Saavedra, José Valbuena, Wilda Olivares, María José Marchant, Andrés Rodríguez, Verónica Torres-Estay, Gonzalo Carrasco-Avino, Leda Guzmán, Francisco Aguayo, Juan Carlos Roa, Alejandro H. Corvalán

**Affiliations:** 1 Advanced Center for Chronic Diseases (ACCDiS), Pontificia Universidad Católica de Chile, Santiago, Chile; 2 UC—Center for Investigational Oncology (CITO), Pontificia Universidad Católica de Chile, Santiago, Chile; 3 Scientific and Technological Bioresource Nucleus (BIOREN), Centro de Estudios Genéticos e Inmunológicos (CEGIN) and Department of Pathology, Universidad de La Frontera, Temuco, Chile; 4 Department of Pathology, Pontificia Universidad Católica de Chile, Santiago, Chile; 5 Instituto de Química, Pontificia Universidad Católica de Valparaíso, Valparaíso, Chile; 6 Department of Pathology, Universidad de Chile, Santiago, Chile; 7 Pathology Department Icahn School of Medicine at Mount Sinai, New York, New York, United States of America; 8 Virology Program, Institute of Biomedical Sciences, Faculty of Medicine, Universidad de Chile, Santiago, Chile; 9 Department of Hematology-Oncology, Faculty of Medicine, Pontificia Universidad Católica de Chile, Santiago, Chile; Vanderbilt University Medical Center, UNITED STATES

## Abstract

Reprimo (RPRM), a downstream effector of p53-induced cell cycle arrest at G2/M, has been proposed as a putative tumor suppressor gene (TSG) and as a potential biomarker for non-invasive detection of gastric cancer (GC). The aim of this study was to evaluate the epigenetic silencing of RPRM gene by promoter methylation and its tumor suppressor function in GC cell lines. Furthermore, clinical significance of RPRM protein product and its association with p53/p73 tumor suppressor protein family was explored. Epigenetic silencing of RPRM gene by promoter methylation was evaluated in four GC cell lines. Protein expression of RPRM was evaluated in 20 tumor and non-tumor matched cases. The clinical significance of RPRM association with p53/p73 tumor suppressor protein family was assessed in 114 GC cases. Tumor suppressor function was examined through functional assays. RPRM gene expression was negatively correlated with promoter methylation (Spearman rank r = -1; p = 0.042). RPRM overexpression inhibited colony formation and anchorage-independent growth. In clinical samples, RPRM gene protein expression was detected in 75% (15/20) of non-tumor adjacent mucosa, but only in 25% (5/20) of gastric tumor tissues (p = 0.001). Clinicopathological correlations of loss of RPRM expression were significantly associated with invasive stage of GC (stage I to II-IV, p = 0.02) and a positive association between RPRM and p73 gene protein product expression was found (p<0.0001 and kappa value = 0.363). In conclusion, epigenetic silencing of RPRM gene by promoter methylation is associated with loss of RPRM expression. Functional assays suggest that RPRM behaves as a TSG. Loss of expression of RPRM gene protein product is associated with the invasive stage of GC. Positive association between RPRM and p73 expression suggest that other members of the p53 gene family may participate in the regulation of RPRM expression.

## Introduction

Gastric cancer (GC) is the third leading cause of cancer-related death and the fifth most common malignancy worldwide [1]. Despite the decreasing incidence of GC, in part due to the recognition of certain risk factors (e.g. *Helicobacter pylori* and environmental factors), it remains one of the most common cancers worldwide and continues to be a clinical challenge [2,3]. Gastric carcinogenesis involves a gradual accumulation of genetic and epigenetic alterations, leading to dysregulation in the expression of oncogenes and tumor suppressor genes (TSGs) [4]. Reprimo (RPRM) is a novel putative TSG [5,6] and associated with gastric carcinogenesis [7]. Moreover we have proposed that methylated RPRM cell-free DNA may be a potential biomarker for the non-invasive detection of GC [8,9].

RPRM is a highly glycosylated protein localized predominantly in the cytoplasm and has been identified as a downstream effector of p53-induced cell cycle arrest at G2/M [10]. Reports suggest that RPRM expression is regulated by two mechanisms, one through DNA methylation at its promoter region [8] and the other by p53 pathway [10]. However, more recent studies have not been able to confirm these findings [6]. On the other hand, the clinical significance of RPRM has been poorly studied [11]. In the present study we evaluated the role of DNA methylation in the regulation of RPRM expression, its clinical significance and its association with members of the p53 tumor suppressor protein family (i.e. p53 and p73). We found dense methylation in the RPRM promoter region, which was associated with loss of expression in GC cell lines. In clinical cases, loss of expression was associated with the invasiveness stage of GC. Furthermore, we observed a positive association between expression of RPRM and p73, suggesting that other members of the p53 gene family might be novel candidates for the regulation of RPRM expression.

## Methods

### Cell Lines and Tissue Samples

Four GC cell lines (AGS, SNU-1, KATOIII and NCI-N87) were purchased from American Type Culture Collection (ATCC) in December 2012 cultured in RPMI-1640 medium (Hyclone) and supplemented with 10% fetal bovine serum (FBS) and maintained at 37°C, 5% CO_2_. Twenty matched tumor and non-tumor adjacent mucosa (NTAM) samples were selected for evaluation of RPRM expression. Six were selected for evaluation of RPRM methylation. A cohort of 114 GC patients was selected for clinicopathological correlations of RPRM gene protein product. Tissue samples from individual cases were classified in accordance to Japanese Research Society for Gastric Cancer recommendations [12]. All cases were recruited from the Instituto Chileno Japonés de Enfermedades Digestivas—Hospital Clínico San Borja Arriarán (ICHJED-HCSBA), Santiago, Chile between 1993–2000 [13] and clinicopathological features are shown in Table 1. This study was approved by the Institutional Review Boards of Pontificia Universidad Católica de Chile (Comité de Ética Científico) and ICHJED-HCSBA (Comité de Ética Científico, Servicio de Salud Metropolitano Central, Santiago, Chile). Written informed consent was obtained from each participant involved in the study.

**Table 1:** Clinicopathological correlations of RPRM in gastric cancer.

		n	Positive n(%)	*p* **	*p* ***
**Total**		**114**			
**Gender**				0.471	0.688
	*Female*	39	13 (33.3)		
	*Male*	72	29 (40.3)		
**Missing cases**		3			
**Age (years)**				0.698	0.470
	*≦61*	45	18 (40.0)		
	*> 61*	66	24 (36.4)		
**Missing cases**		3			
**Location**				0.533	0.182
	*Cardia*	42	19 (45.2)		
	*Middle*	28	10 (35.7)		
	*Antral*	37	11 (29.7)		
**Missing cases**		7			
**Histology**				0.732	0.973
** **	*Diffuse*	50	22 (38.6)		
** **	*Intestinal*	57	18 (36.0)		
**Missing cases**		7			
**Tumor size (cm)**				0.560	0.431
	*≦4*.*9*	36	15 (41.7)		
	*>5*.*0*	67	24 (35.8)		
**Missing cases**		11			
**Lymph node metastasis**				0.037*	0.217
	*Negative*	40	20 (50.0)		
	*Positive*	67	20 (29.9)		
**Missing cases**		7			
**Pathological staging**				0.006*	0.020*
	*I*	20	13 (65.0)		
	*II-IV*	94	30 (31.9)		
**Missing cases**		0			

*p<0.05

** Pearson Chi-Square Test (categorical variables)

*** Logistic regression (Multivariate analyses)

### Tissue microarray and Immunohistochemistry

Tissue microarrays (TMA) were done by using a Manual Tissue Array II instrument (Beecher Instruments) as previously described [14,15]. Core sections (4μm) were subjected to immunostaining by Vectastain Elite Kit R.T.U (Vector Labs), according to manufacturer’s instructions. Antibodies used in this study were RPRM (38–50, Sigma-Aldrich), p53 (clone 318-6-11, Dako Denmark) and p73 protein α (clone 24, Novacastra). Results of immunostaining in whole tumor and NTAM as well as TMA sections were considered positive for RPRM if >30% of epithelial cells showed cytoplasmic staining, >10% nuclear staining for p53, or >10% nuclear/cytoplasmic staining for p73. Evaluation of immunohistochemical staining was performed independently by two pathologists (JV & GC) who were blinded to clinical data.

### Bisulfite sequencing and quantitative RT-PCR Expression Analysis

DNA from GC cell lines was extracted using TRIzol reagent (Life Technologies) according manufacturer’s protocol, followed by bisulfite conversion using the EZ DNA methylation Gold kit (Zymo Research). RPRM promoter was amplified from bisulfite-treated DNA, using primers RPRM_B_FW 5’-TTGTAAAAGTAAGTAATAAAAAGTAAG-3’ and RPRM_B_RV 5’-CTACTATTAACCAAAAACAAAC-3’, allowing the detection of 52 CpG sites within a 509-bp promoter region. PCR products were purified with QIAXEN II gel extraction kit (QIAGEN) and ligated into pGEM-T vector. The ligation product was used to transform competent *E*. *coli* Top10 cells, according to the procedure described by Inoue et al. [16]. The transformants were selected on LB agar plates supplemented with ampicillin (100 μg/mL), X-Gal (50 mg/mL) and IPTG (100 mM). The resulting white colonies were assessed by colony-PCR. Positive clones were grown on liquid medium (LB/ampicillin) until late exponential phase (OD600 = 1) and DNA plasmids were purified using Pure Yield miniprep system kit (Promega). The RPRM promoter region was sequenced using universal M13 (W 5’-GTAAAACGACGGCCAG-3’ and RV 5’-CAGGAAACAGCTATGAC-3’) by Macrogen (http://dna.macrogen.com). BiQ analyzer software was used for the analysis of sequenced clones. All positive pGEM-T clones were grown on LB/ampicillin medium and stored at -80°C (in 14% glycerol). Total RNA was extracted using TRIzol reagent (Invitrogen) and cDNA was synthesized using iScript cDNA Synthesis kit according to the manufacturer’s instructions (Biorad). Subsequently, cDNA was used for each PCR reaction with each primer pair. The RPRM specific primers are: RPRM_FW 5’-GAGCGTAGCCTGTACATAATGC-3’ and RPRM_RV 5’-CCTTCACGAGGAAGTTGATCAT-3’. Real time RT–PCR analysis was performed using LightCycler Fast Start DNA MasterPlus SYBR Green I (Roche) in a LightCycler 1.5 Real-Time Detection System (Roche). Relative quantitative analysis normalized to RPL-30 was conducted via the comparative cycle threshold method [17]. The RPL-30 specific primers are: RPL-30_Fw 5’-ACAGCATGCGGAAAATACTAC-3’ and RPL30_RV 5’-AAAGGAAAATTTTGCAGGTTT-3’.

### Validation of RPRM antibody by Immunoblot and Immunofluorescence

3x10^5^ AGS cells were transiently transfected with pCMV6/RPRM or pCMV6 (Origene) empty vector, using Lipofectamine 2000 (Invitrogen), according to manufacturer’s protocol. Forty-eight hours after transfection, cells were incubated for 24 h in RPMI1640 with 10% FBS in the presence or absence of N-glycosylation inhibitor Tunicamicyn (10 ng/mL). Cells were harvested and whole-cell lysates were extracted using Triton buffer (Tris-HCl 50mM pH7.5, NaCl 0.1M, 0.5% Triton X-100) with Protease Inhibitor Cocktail Kit (P8340, Sigma Aldrich) and Phosphatase Inhibitor Cocktail Kit (sc-45045, Santa Cruz Biotechnology). Protein concentrations were determined using Quick Start Bradford Reagent (Bio-Rad). Fifty μg of protein were separated on SDS-PAGE 4% to 12%. Proteins on the gels were electroblotted to polyvinylidene difluoride membranes using the mini transblotter system (Bio-Rad, Hercules, CA). The polyvinylidene difluoride membranes were blocked in 5% milk in Tris-buffered saline/Tween 20 (TBST) for 1 h. Primary antibody anti-RPRM (38–50, Sigma-Aldrich) was diluted 1:1000 in TBST/3% BSA/ and the membranes were incubated in primary antibodies at 4°C overnight. Membranes were washed three times in TBST for 10 min each. Anti-rabbit peroxidase-conjugated secondary antibody (Santa Cruz) was diluted 1:2000 in TBST and incubated on the membranes for 1 h at room temperature. The membranes were washed as above and visualized using SuperSignal West Pico Chemiluminescent Substrate (Pierce) according to the manufacturer’s protocol. To evaluate the cellular location of RPRM, immunofluorescence assay was performed. AGS cells transiently transfected with pCMV6-RPRM or pCMV6 were grown on coverslips. The transiently transfected AGS cells were fixed in 4% paraformaldehyde for 20 min at room temperature and then washed three times in Phosphate-buffered saline (PBS). Cells were permeabilized in 0.1% Triton-X-100 (Sigma–Aldrich) for 10 min, blocked in 3% BSA for 1 h at room temperature, and subsequently labeled with an anti-RPRM antibody (1:1000, 38–50, Sigma-Aldrich). After washing with PBS, cells were incubated with an Alexa Fluor 488-conjugated antibody (1:200, Molecular Probes, Invitrogene) for 1 h at room temperature. Images were acquired by fluorescence laser scanning confocal microscopy (see Supporting Information S1 and S2 Figs).

### Cell culture and transfection

For stable transfection experiments, AGS cells were plated at 3x10^5^ cells/100-mm culture dish and transfected after 24 h with pCMV6-RPRM or pCMV6 (Origene) empty vector using Lipofectamine 2000 (Invitrogen), according to the manufacturer’s protocol. Forty-eight hours after transfection, cells were cultured under the same conditions with G418 (500g/mL). Culture media was changed every 24 h for 14 days.

### Colony formation assay

For colony formation assay, 200 cells were stably transfected with pCMV6-RPRM or pCMV6 empty vector. Cells were cultured in RPMI-1640 medium (Hyclone) and supplemented with 10% FBS. Culture media was changed every 24 h. Colonies were stained using 0.4% crystal violet (Sigma) in 50% methanol, 21 days after initial seeding, and counted at 20 images taken under inverted microscope (Evos XL Core Cell imaging System, Life Technologies). Each transfection was carried out in triplicate. In addition, replicate experiments were carried out to obtain further clones for expression analysis.

### Anchorage-independent growth assay

Anchorage-independent cell growth was analyzed by plating 1% agarose containing 2x10^5^ cells stable transfected with pCMV6-RPRM or pCMV6 empty vector in 6-well plates. Cells were fed weekly by overlying fresh soft-agar solution, and colonies were photographed after 4 weeks of incubation. The experiment was carried out in triplicate.

### Statistical Analysis

Spearman correlation coefficient was calculated to analyze the correlation between RPRM methylation and gene expression levels. Categorical variables were analyzed by Pearson Chi-Square Test (two-sided), the p-value was corrected using a logistic regression analysis. Continuous variables were analyzed using Student’s *t* test and data was expressed as mean ± SD. Kappa test was used to analyze the correlation between the RPRM expression and p73 expression. Statistical analyses were performed using SPSS version 17.0 (SPSS Inc., Chicago, IL) statistical package and GraphPad Prism5 software (La Jolla, CA, United States). All of the values were two-tailed, except for Spearman rank correlation. Statistical significance was defined as p < 0.05.

## Results

### RPRM methylation and expression in gastric cancer cell lines

**Fig 1:**
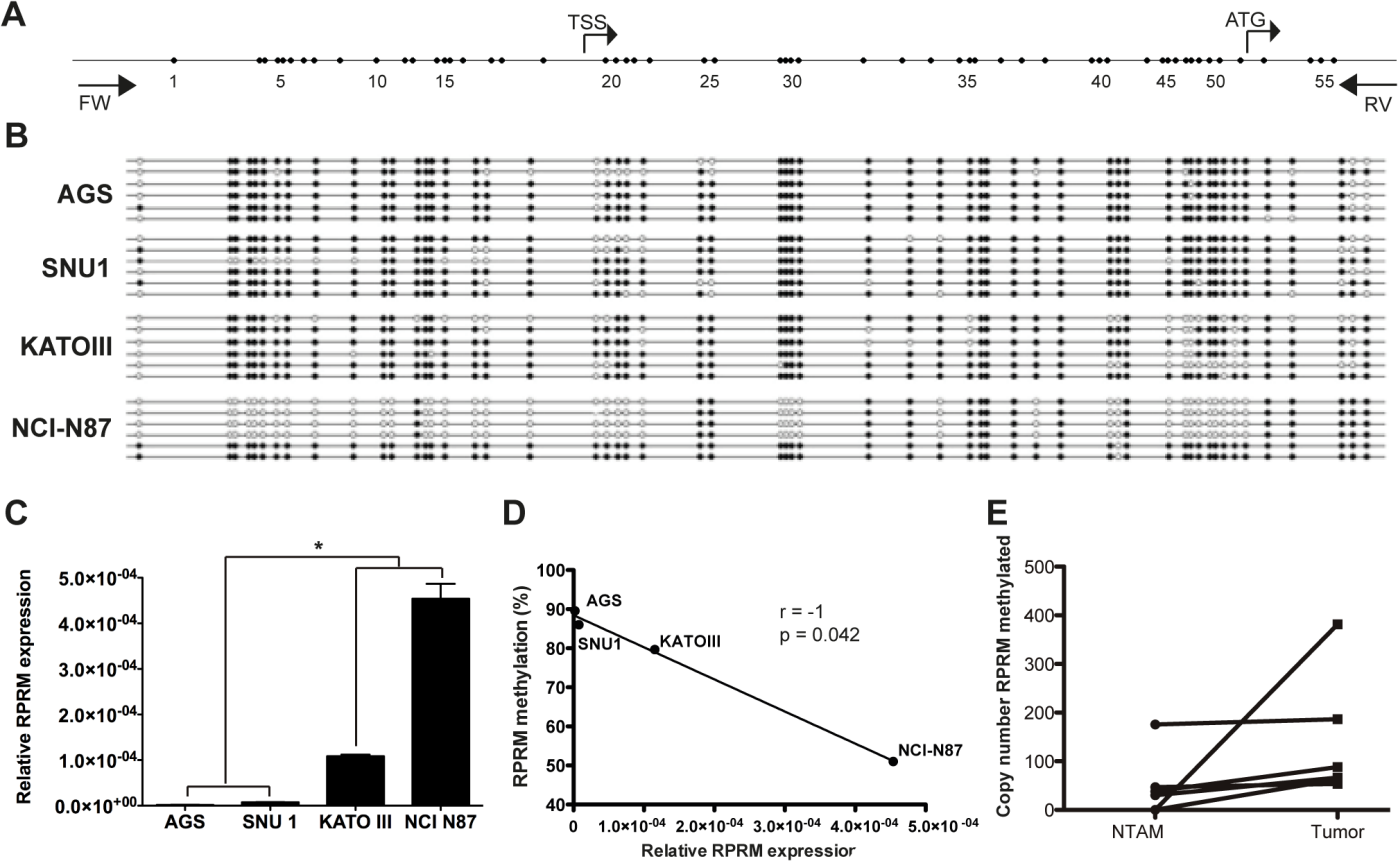
Regulation of RPRM expression by methylation of its promoter region. A) RPRM promoter region analyzed by bisulfite quantification. B) DNA Bisulfite sequencing in gastric cancer cell lines. C) Relative expression of RPRM in four gastric cancer cell lines ns: no significative; * p<0.01; ANOVA test One-way. D) Spearman rank correlation between RPRM expression and promoter methylation status of RPRM. E) RPRM methylation in tumor and non-tumor adjacent mucosa (NTAM) tissues, higher methylation levels in tumor tissues are observed in comparison to NTAM in all six paired GC cases.

Previously, we and others have reported that RPRM expression can be restored by the demethylating agent 5-aza-2-deoxycytidine (See Supporting Information S3 and S4 Figs) [5,8,18]. However RPRM methylation has been weakly correlated with its expression [18]. To further assess the association between RPRM promoter methylation and gene expression, the complete promoter region of RPRM gene was analyzed by DNA bisulfite sequencing in four GC cell lines (AGS, SNU-1, KATO III and NCI-N87) (Fig 1A). Thus, the methylation status of 52 CpG sites, located between -207 and +302 nucleotides relative to the transcription start site (TSS), were analyzed. This analysis shows dense methylation in cell lines AGS and SNU-1 (89.6% and 86%, respectively), in comparison to KATO III and NCI-N87 (79.7% and 51.8%, respectively)(p = 0.0002) (Fig 1B). Next, level of RPRM expression was determined by quantitative RT-PCR. Low RPRM gene expression was observed in AGS and SNU-1 compared to KATO III and NCI-N87 cell lines (p<0.0001) (Fig 1C). By Spearman’s correlation analysis revealed significant negative correlation was found between methylation density and RPRM gene expression (r = -1; p = 0.042) (Fig 1D). Taken together, these findings suggest that methylation of RPRM promoter region plays a critical role in the regulation of RPRM expression.

### RPRM methylation and expression in gastric cancer tissues

**Fig 2:**
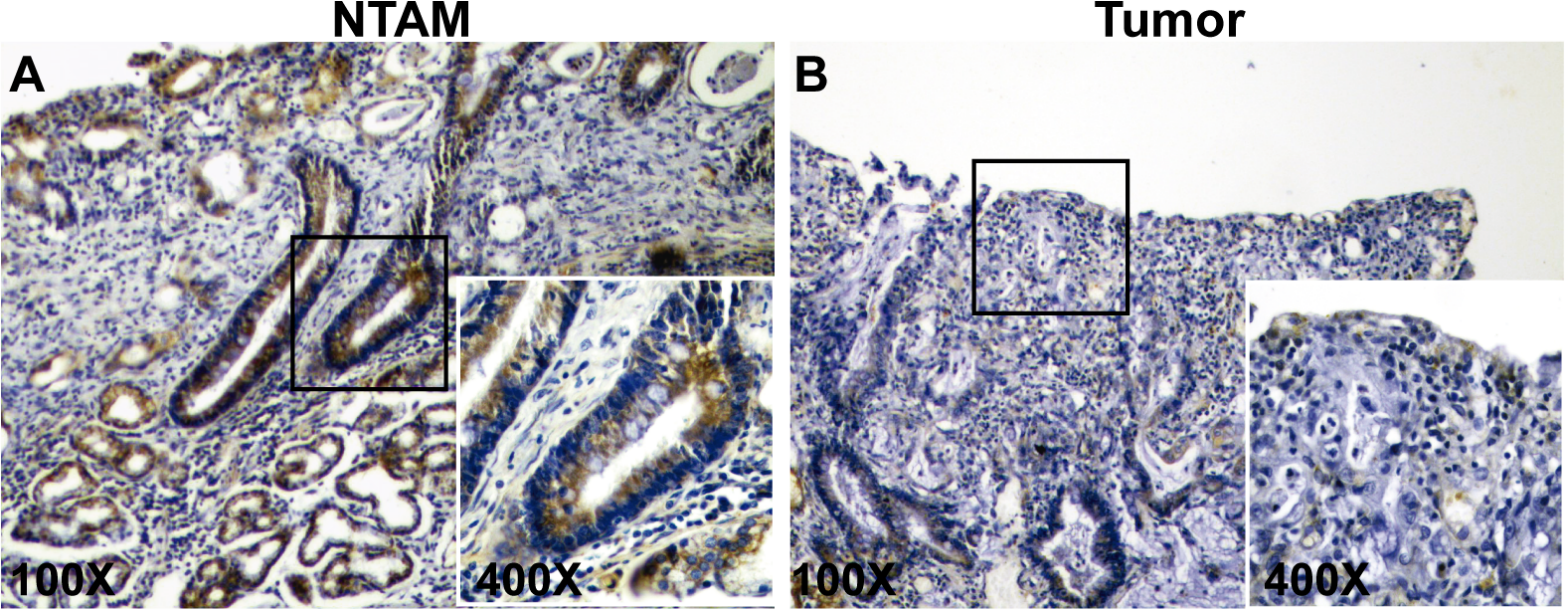
Immunohistochemical staining for RPRM in gastric cancer. A) Representative example of non-tumor adjacent mucosa (NTAM) showing positive cytoplasmic staining of RPRM in more than 30% of cells. B) Representative example of tumor (T) gastric cancer sample showing negative staining of RPRM.

To corroborate previous data in GC tissues, RPRM methylation levels were determined in 6 paired tumor and NTAM samples. Results indicate higher methylation levels in tumor tissues compared to NTAM tissues (See Fig 1E). To investigate the expression of RPRM gene protein product in GC tissues IHC analysis was performed. Positive RPRM expression was mainly observed in the cytoplasm of gastric epithelial cells. RPRM gene protein expression was detected in 75% (15/20) of NTAM tissues samples. However only 25% (5/20) of tumor samples showed expression of RPRM gene protein product (Fig 2). These differences were highly significant (p = 0.001) and suggest that expression of RPRM is lost in GC tissues. To evaluate the clinical significance of this loss of RPRM expression a cohort of 114 GC cases was evaluated. Loss of RPRM expression was found in 62% (71/114) of GC cases. Clinicopathological correlations of RPRM expression are shown in Table 1. Progression from stage I GC to stages II-IV (p = 0.006) and lymph node metastasis (p = 0.037) were significantly associated with loss of expression of RPRM gene protein product. However, logistic regression analysis shows that only progression from stage I to II-IV is significantly associated with loss of RPRM gene protein product (p = 0.020).

### Functional assay of RPRM in gastric cancer (colony formation and anchorage-independent growth)

**Fig 3:**
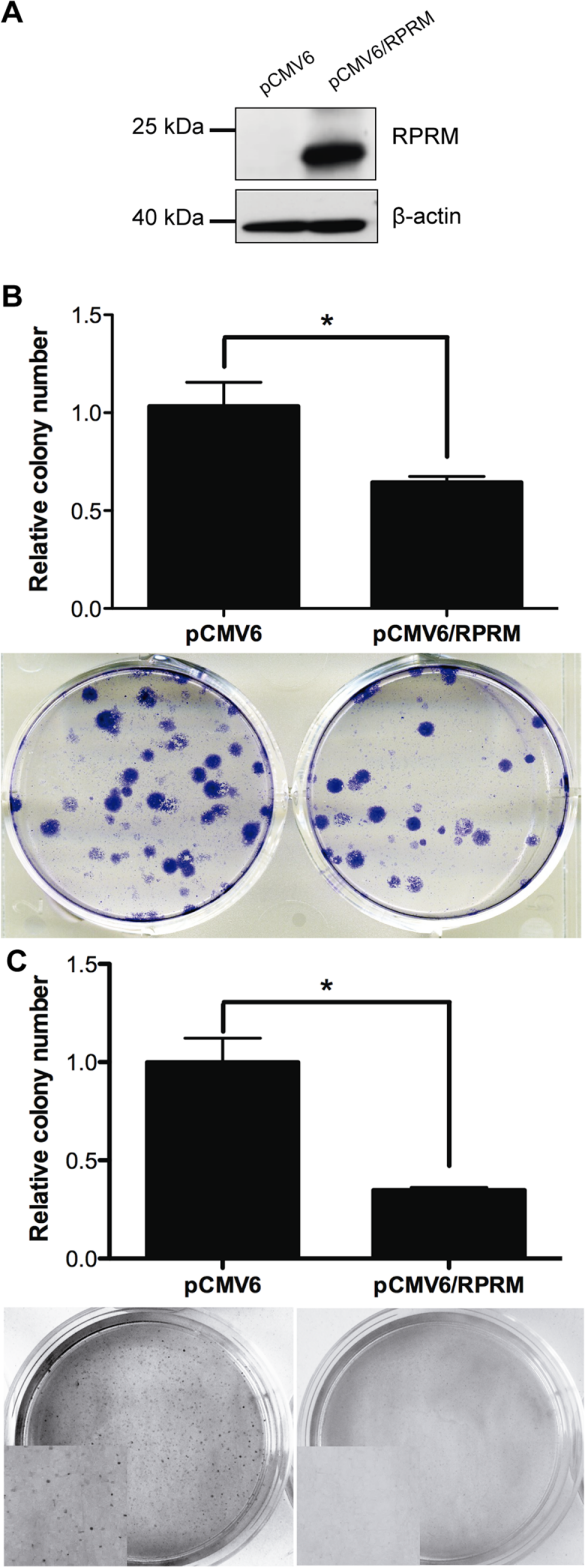
Overexpression of RPRM reduces colony formation and anchorage-independent growth *in vitro*. A) Western blot of AGS cells with overexpression of RPRM (pCMV6/RPRM) and empty control (pCMV6). B) Overexpression of RPRM in AGS cells (pCMV6/RPRM) resulted in a significant reduction of *in vitro* colony formation compared with AGS cell line transfected with pCMV6 empty vector (pCMV6). Each experiment was carried out in triplicate. There was a statistically significant reduction of colony formation in overexpression experiments (*p < 0.05). Below each graph are representative plates showing reduction of colonies after gene overexpression Error bars; SD. C) Overexpression of RPRM in AGS cells (pCMV6/RPRM) resulted in a significant reduction of *in vitro* anchorage-independent colony formation compared with AGS cell line transfected with pCMV6 empty vector (pCMV6). Each experiment was carried out in triplicate. There was a statistically significant reduction of colonies formation in the overexpression experiments (*p < 0.05). Below each graph are representative plates showing reduction of colonies after gene overexpression Error bars; SD

As the loss of RPRM expression was associated in GC and mainly with the progression from stage I to II-IV, a tumor suppressor role of RPRM might be plausible. Therefore, functional assays such as *in vitro* colony formation and anchorage-independent growth assays were performed in AGS cell transfected with pCMV6/RPRM (Fig 3A). Non-expressing AGS cell lines transfected with RPRM expression plasmids showed a significantly reduced number of colonies in comparison to pCMV6- empty vector-transfected cell lines transfected with pCMV6-empty vector (p<0.05) (Fig 3B). The effect of RPRM overexpression on anchorage-independent growth in a soft agar colony formation assay was also assessed in stable transfected AGS cell line clones. Colonies were counted after initial seeding and incubation in soft agar for 4 weeks. Cells transfected with empty vector showed robust colony growth, by number and size of colonies. This was greatly reduced when RPRM was re-expressed in AGS cells (Fig 3C).

### Association of RPRM expression and p53/p73 tumor suppressor protein family in gastric cancer

RPRM has been defined as a p53-mediated gene in embryonic fibroblast cells [18]. However, it has been reported that regulation of RPRM expression might be independent from p53 gene status in neuronal cells treated with copper [19]. On the other hand p73 can activate the transcription of p53-responsive genes [20]. Considering this setting, we evaluated the expression of p53 and other member of the p53 protein family, p73 TSG. Expression of p53 was located in the nuclei of gastric mucosa cells. Positive p53 expression was detected in 23.5% (20/114) of GC cases. Only tumor size was associated with the expression of p53 (p = 0.035, see Table 2). Expression of p73 was located in the cell nuclei and cytoplasm of epithelial cells. Positive p73 expression was found in 30.6% (34/114) of GC samples. Interestingly, significant clinicopathological correlations of p73 expression were associated with invasive stage (stage I 55.6%; 10/18 to stages II-IV 25.8%; 24/93, p = 0.012) and lymph node metastasis (p = 0.038) (Table 2). Since losses of expression of RPRM and p73 gene protein products have similar clinicopathological correlations, we evaluated association between expressions of levels of both proteins. To this end cases were separated into four groups according to the expression of RPRM and p73 protein products. The analysis showed that 22 out 111 GC cases were positive for both RPRM and p73 proteins, whereas 57 out 111 were negative. This association was statistically significant (p<0.0001) with a kappa value of 0.363 (Table 3).

**Table 2:** Clinicopathological correlations of p53/p73 tumor suppressor protein family in gastric cancer.

		p53			p73		
		n	Positive n(%)	*p*	n	Positive n(%)	*p*
** **		** **	** **	** **	** **	** **	** **
**Total**		**114**	** **	** **	**114**	** **	** **
**Age (years)**				0.195			0.539
** **	*<61*	32	5 (15.6)		44	12 (27.3)	
** **	*≥ 61*	50	14 (28.0)		64	21 (32.8)	
**Missing cases**		32			6		
**Gender**				0.979			0.253
** **	*Female*	30	7 (23.3)		38	9 (23.7)	
** **	*Male*	52	12 (23.1)		70	24 (34.3)	
**Missing cases**		32			6		
**Tumor size (cm)**				0.035*			0.084
** **	*<4*,*9*	24	9 (37.5)		36	15 (41.7)	
** **	*>5*,*0*	51	8 (15.7)		64	16 (25.0)	
**Missing cases**		39			14		
**Location**				0.676			0.849
** **	*Cardia*	31	8 (25.8)		42	14 (33.3)	
** **	*Middle*	22	6 (27.3)		26	7 (26.9)	
** **	*Antral*	26	4 (15.4)		36	10 (27.8)	
**Missing cases**		36			8		
**Histology**				0.432			0.377
** **	*Diffuse*	41	8 (19.5)		49	13 (26.5)	
** **	*Intestinal*	37	10 (27.0)		55	19 (34.5)	
**Missing cases**		36			10		
**Pathological staging**				0.324			0.012*
** **	*T1*	15	5 (33.3)		18	10 (55.6)	** **
** **	*T2+3+4*	70	15 (21.4)		83	24 (25.8)	** **
**Missing cases**		29			3		** **
**Lymph node metastasis**				0.583			0.038*
** **	*Negative*	33	9 (27.3)		43	18 (41.9)	
** **	*Positive*	50	11 (22.0)		65	15 (23.1)	
**Missing cases**		35			6		

*p<0.05, Pearson Chi-Square Test (categorical variables)

**Table 3:** Kappa value for RPRM and p73 expression in gastric cancer.

	RPRM			Value	p	Kappa
		*Positive*	*Negative*			
**p73**	*Positive*	22 (64.7%)	12 (35.3%)	15.043	<0.0001	0.363
	*Negative*	20 (26.0%)	57 (74.0%)			

*p <0.05, x^2^ test and Kappa correlation

## Discussion

RPRM is a highly glycosylated cytoplasmic protein, initially identified as a downstream effector of p53-induced cell cycle arrest at G2/M. Previously, it has been proposed that RPRM is silenced by promoter methylation, although it has been weakly correlated with its expression [5,8]. In addition, RPRM has been proposed as a putative tumor suppressor gene in renal cell carcinoma and pituitary tumors [5,6]. In this study, we demonstrated that RPRM gene expression is strongly associated with its promoter region methylation status, suggesting epigenetic silencing of RPRM gene expression. In addition, we show that RPRM gene protein product was decreased in GC tissue compared with noncancerous gastric mucosa. These results are similar to that of Luo *et al*. [11], who reported loss of RPRM in GC when compared to gastric ulcer tissue samples. Furthermore, our finding regarding loss of expression of RPRM in the transition from stage I to stages II-IV is clinically relevant. Luo *et al*. [11] reported a similar finding, but in a small number of stage I GC cases (n = 15). These findings may have clinical significance as a predictive factor for the progression of GC. Accordingly with this loss of RPRM expression in the progression of GC, our functional assays (colony formation and anchorage-independent growth) also proposed a putative tumor suppressor role for RPRM in GC.

Although p53 was initially described as a RPRM inductor [10], clinical studies performed up to date have not been able to confirm such finding [6,18]. Here, we identified that loss of RPRM expression correlates with loss of expression of another member of p53 family, p73. Although p73 TSG is expressed at a very low level in normal human tissues [21], is overexpressed in many different human cancers such as breast, neuroblastoma, lung, esophagus, stomach, colon, bladder and ovary [14,22]. In addition, it has been reported that p73 can activate the transcription of p53-responsive genes, including p21Waf1/Cip1, bax, mdm2, cyclin-G, GADD45 and IGFBP3 [20]. Thus, based on our findings, we propose that RPRM expression could be regulated by p73 in a p53-independent manner.

In summary, our data suggest that epigenetic silencing of RPRM gene by promoter methylation is associated with loss of RPRM expression and accordingly, functional assays proposed a putative tumor suppressor role of RPRM in GC. In clinical samples, RPRM is lost at invasive stages of GC and its expression correlates with that of p73 suggesting that other members of the p53 gene family may participate in the regulation of RPRM expression. Further research is warranted to characterize the role of RPRM in the progression of GC and validate the biological regulation of RPRM by p73.

## Supporting Information

S1 Data Data underlying the study. (ZIP)

S1 Fig Effects of Tunicamicyn on RPRM protein. RPRM is a highly glycosylated cytoplasmic protein visualized to 25 kD by Western blot, the glycosylation inhibitor tunicamycin (TK) displaces the 25 kD RPRM band to 15 kD in AGS cells with RPRM overexpression (pCMV6/RPRM) (RPRM predicted size 12 kD). Cells were incubated for 24 h in RPMI1640 with 10% FBS in the presence or absence of the inhibitor of N-glycosylation tunicamicyn (10 ng/mL). RPRM expression was determined by Western blotting using a RPRM polyclonal antibody (upper panel, dilution 1:1000, Sigma-Aldrich). The expression of β-actin (lower panel, dilution 1:2000, Santa Cruz) represents protein loading.
(TIF)

S2 Fig Localization of RPRM expression in overexpressing AGS cell line Immunofluorescence of RPRM in gastric cancer AGS cell line transfected with pCMV6 vector encoding RPRM. 24 h post-transfection cells were fixed in paraformaldehyde 4% and incubated with anti-RPRM-rabbit (1:1000, 38–50, Sigma-Aldrich) and secondary antibody Alexa Fluor-488 (1:200, Molecular Probes, Invitrogene). A positive expression of RPRM (GREEN) is mainly seen in the cytoplasm. Images were captured at using Axio Vision4 multichannel software in fluorescence microscope Axio Scope.A1- Zeiss.
(TIF)

S3 Fig RPRM expression is silenced by promoter methylation. A) RT-PCR analysis of RPRM mRNA expression in AGS gastric cancer cell line with and without the DNA methylation inhibitor 5-Azacytidine (1 uM for 72 hrs). GAPDH was used as a control. B) Amplification of RPRM by Methylation Specific PCR in AGS gastric cancer cell line. Amplification of methylated MYOD1 was used as control. AGS cell line was methylated in the promoter region. NC: negative control.
(TIF)

S4 Fig RPRM expression is silenced by promoter methylation (original gel). A) RT-PCR analysis of RPRM mRNA expression in AGS gastric cancer cell line with and without the DNA methylation inhibitor 5-Azacytidine (1 uM for 72 hrs). GAPDH was used as a control. B) Amplification of RPRM by Methylation Specific PCR in AGS gastric cancer cell line. Amplification of methylated MYOD1 was used as control. AGS cell line was methylated in the promoter region. NC: negative control.
(TIFF)
