# Supplementary figures and images for: Loss of Expression of Reprimo, a p53-induced Cell Cycle Arrest Gene, Correlates with Invasive Stage of Tumor Progression and p73 Expression in Gastric Cancer
Source: PLoS One. 2015 May 8;10(5):e0125834. doi: 10.1371/journal.pone.0125834 (PMC4425545; doi:10.1371/journal.pone.0125834)

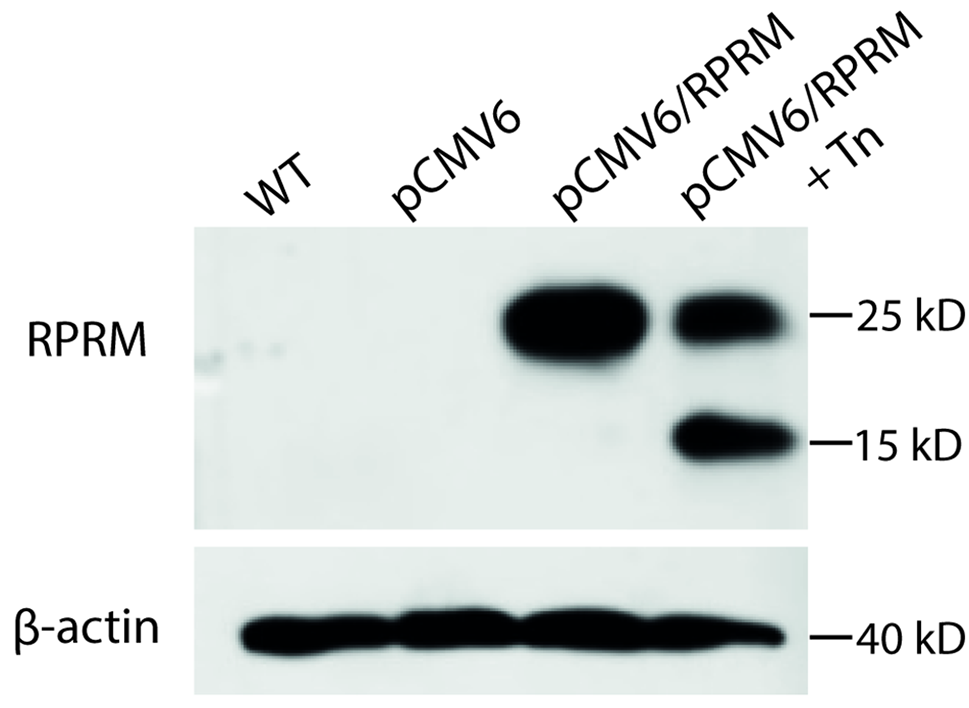

Supplement: S1 Fig — RPRM is a highly glycosylated cytoplasmic protein visualized to 25 kD by Western blot, the glycosylation inhibitor tunicamycin (TK) displaces the 25 kD RPRM band to 15 kD in AGS cells with RPRM overexpression (pCMV6/RPRM) (RPRM predicted size 12 kD). Cells were incubated for 24 h in RPMI1640 with 10% FBS in the presence or absence of the inhibitor of N-glycosylation tunicamicyn (10 ng/mL). RPRM expression was determined by Western blotting using a RPRM polyclonal antibody (upper panel, dilution 1:1000, Sigma-Aldrich). The expression of β-actin (lower panel, dilution 1:2000, Santa Cruz) represents protein loading. (TIF) [file pone.0125834.s002.tif]

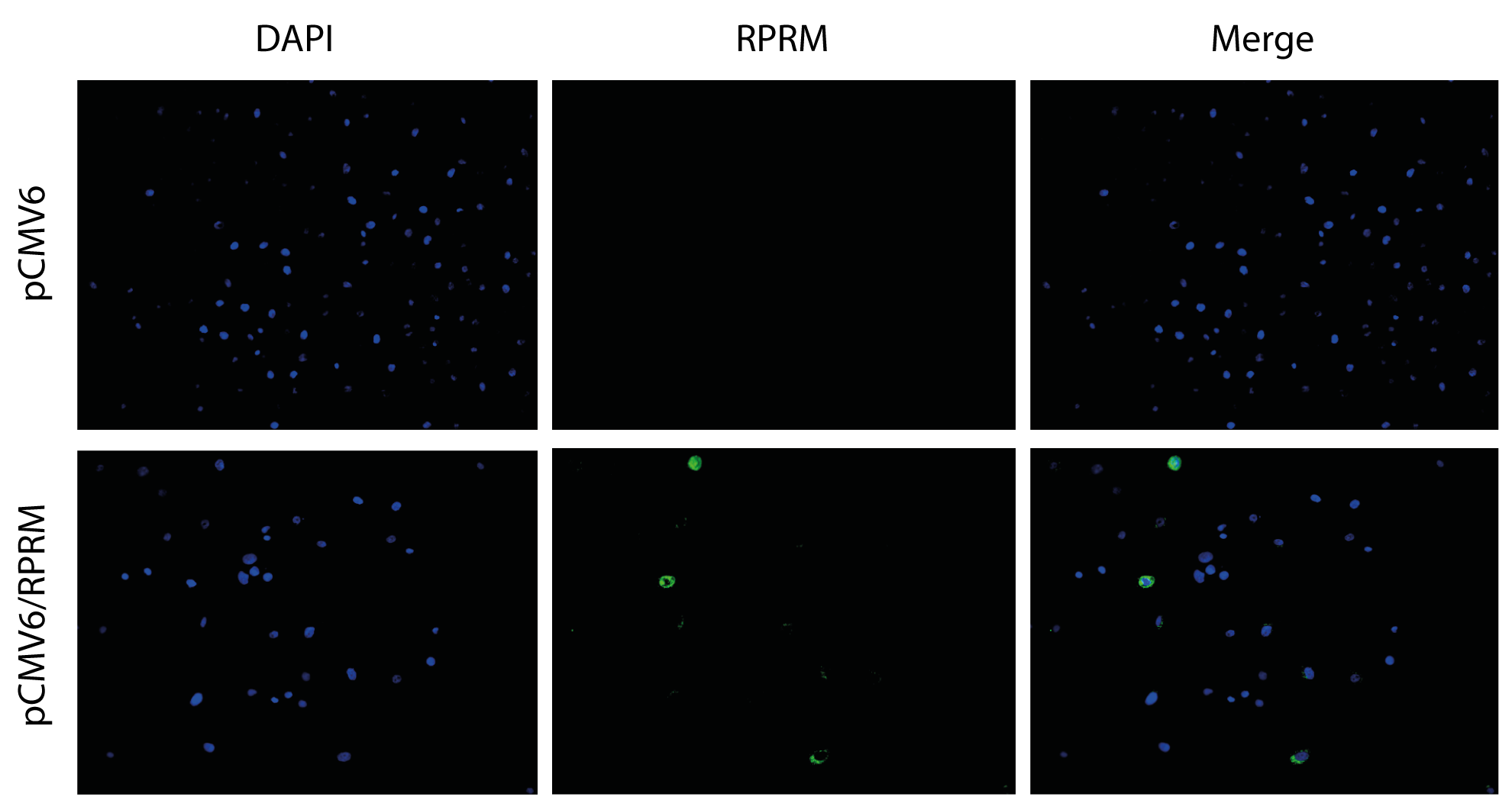

Supplement: S2 Fig — 24 h post-transfection cells were fixed in paraformaldehyde 4% and incubated with anti-RPRM-rabbit (1:1000, 38–50, Sigma-Aldrich) and secondary antibody Alexa Fluor-488 (1:200, Molecular Probes, Invitrogene). A positive expression of RPRM (GREEN) is mainly seen in the cytoplasm. Images were captured at using Axio Vision4 multichannel software in fluorescence microscope Axio Scope.A1- Zeiss. (TIF) [file pone.0125834.s003.tif]

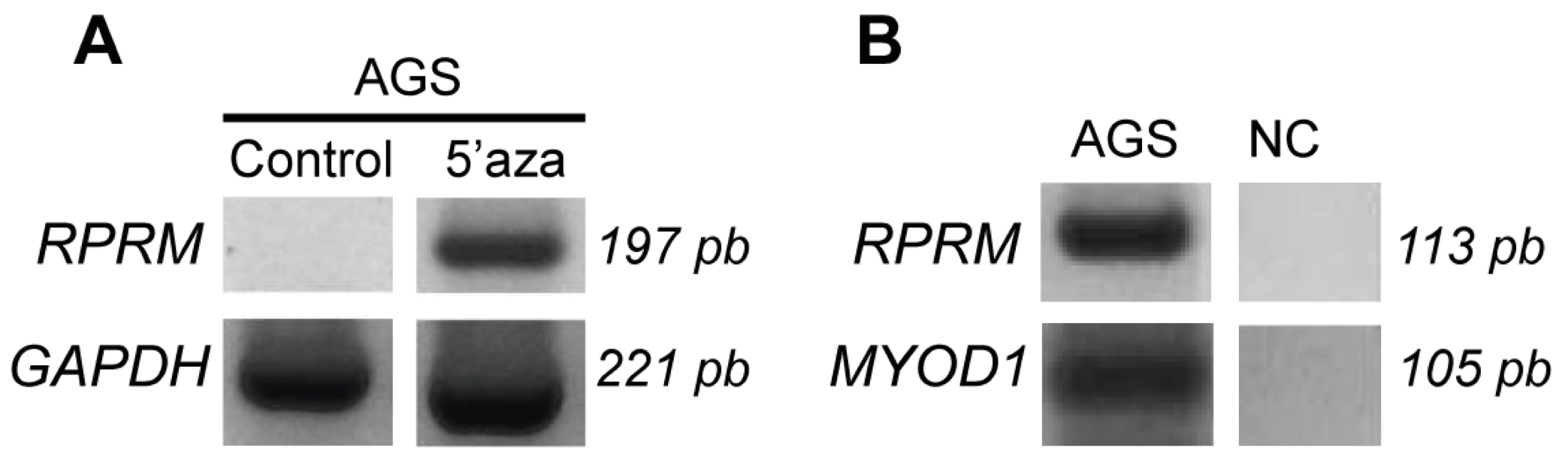

Supplement: S3 Fig — A) RT-PCR analysis of RPRM mRNA expression in AGS gastric cancer cell line with and without the DNA methylation inhibitor 5-Azacytidine (1 uM for 72 hrs). GAPDH was used as a control. B) Amplification of RPRM by Methylation Specific PCR in AGS gastric cancer cell line. Amplification of methylated MYOD1 was used as control. AGS cell line was methylated in the promoter region. NC: negative control. (TIF) [file pone.0125834.s004.tif]

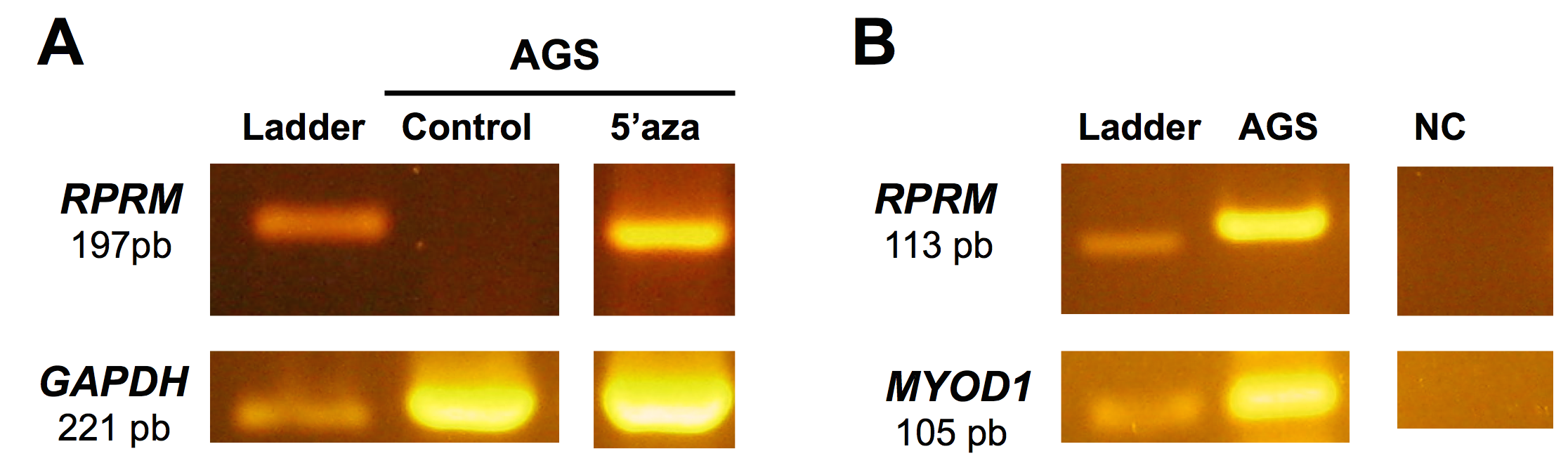

Supplement: S4 Fig — A) RT-PCR analysis of RPRM mRNA expression in AGS gastric cancer cell line with and without the DNA methylation inhibitor 5-Azacytidine (1 uM for 72 hrs). GAPDH was used as a control. B) Amplification of RPRM by Methylation Specific PCR in AGS gastric cancer cell line. Amplification of methylated MYOD1 was used as control. AGS cell line was methylated in the promoter region. NC: negative control. (TIFF) [file pone.0125834.s005.tiff]
